# Ten simple rules for scientists: Improving your writing productivity

**DOI:** 10.1371/journal.pcbi.1006379

**Published:** 2018-10-04

**Authors:** Todd C. Peterson, Sofie R. Kleppner, Crystal M. Botham

**Affiliations:** 1 Department of Neurology & Neurological Sciences, Stanford University, Stanford, California, United States of America; 2 Office of Postdoctoral Affairs, Stanford University, Stanford, California, United States of America; 3 Stanford Biosciences Grant Writing Academy, Stanford University, Stanford, California, United States of America; Dassault Systemes BIOVIA, UNITED STATES

## Introduction

… As a scientist, you are a professional writer. Writing is as important a tool in your toolbox as molecular biology, chemical analysis, statistics, or other purely “scientific” tools. Some of these tools allow us to generate data; others to analyze and communicate results. Writing is the most important of the latter. Because it forms the bridge to your audience, it can act as the rate-limiting step that constrains the effectiveness of all other tools.—Joshua Schimel [1]

Science requires communicating new and exciting findings to diverse audiences. Written communication is especially critical for our success as scientists because we must write to receive degrees (e.g., dissertations), share our discoveries (e.g., manuscripts and abstracts for professional meetings), request funding (e.g., grants, contracts), etc. The process of writing can also refine our research because good writing is an iterative process, with feedback leading to new ideas and experimental follow-up. In reality, we often postpone working on communication until we feel ready or a deadline is imminent, which diminishes our ability for clear writing. We often procrastinate sharing our writing because we consider our audience to be only the peer reviewers of grants and manuscripts. We neglect the opportunity to use perhaps the most important audience of all: ourselves. The best way to engage with ourselves is to develop a strong and sustained writing practice.

Writing every day, even for a short time, improves our thinking and our productivity as scientists. It provides time and space for reflection, allowing new ideas to mature, and maintains perspective on challenging work. We agree with Scott Montgomery that “… ‘clear thinking can emerge from clear writing.’ Imposing order by organizing and expressing ideas has great power to clarify. In many cases, writing is the process through which scientists come to understand the real form and implications of their work” [2]. However, even with the best of intentions, it is easy to postpone writing (Box 1). We believe that establishing a writing practice must be a deliberate act. We further believe that the pay-off for establishing this practice will be found in increased productivity and impact. Here, we outline 10 simple rules for improving your writing productivity, which will also enhance your thinking as a scientist.

**Box 1. Hurdles to writing that all scientists can face**

Types of Writing Resistance

- Evaluation: “This draft stinks.”
- Inspiration: “I don’t have a good idea yet.”
- Motivation: “I just don’t feel like it.”
- Optimization: “I need to make this sentence perfect.”
- Procrastination: “I will start working on it tomorrow.”
- Separation: “I need a lot of time in a quiet place to write.”
- Temptation: “My lab bench is really disorganized; it needs to be cleaned now.”

## Rule 1: Define your writing time

The essential key for writing is to write regularly—like it or not—great ideas come often by writing; releasing the subconscious—waiting for inspiration and ideas will not work, but it does help to have a notebook with you all the time for sudden brainstorms or inspiration.—Dr. Robert Marc Friedman [3]

Commit yourself to writing daily or at least three to four times per week by defining the time when you will write. Pick a bitesize chunk of time where you are unlikely to have conflicts, so that this time is protected for writing and nothing else. For example, if writing right before bed results in a conflict with exhaustion, choose a time earlier in the day. Setting aside just 15 to 30 minutes each day may be sufficient because even short amounts of time can enable meaningful increases in your productivity. As you set your initial goal, consider the SMART criteria: specific, measurable, action-oriented, reasonable, time-bound [4]. For example: “I am going to write from 8:30 AM to 9:30 AM on weekdays.”

Write down your goal on a sticky note, and place it where you will have a daily reminder of your goal—attach it to your coffee grinder or stick it on your computer screen or near your pipettes. Block off the time on your calendar as another permanent reminder of your commitment to writing. You might also use a calendar reminder or an alarm on your phone to catch your attention when it is time to start writing. Then, when you are more comfortable with your writing schedule, consider building up the time you spend writing.

## Rule 2: Create a working environment that really works

Where are you most likely to settle in and write without external distractions? Some people work best in a café or on a flight. Others need complete silence and are most effective working in the library. Once you have identified your preferred writing space, learn to manage distractions. If you find yourself focused on that email you forgot to send, or if finding an antibody for your upcoming experiment suddenly seems urgent, you may want to start a running “to do” list and address it at the conclusion of your writing time. Resist the urge to write a quick email or other small task because it creates further opportunities to procrastinate. Unavoidable interruptions can be managed by saying, “I am committing this time to writing, can I contact you when I am done?” If you have difficulty ignoring the siren call of new emails or the internet, turn it off for the duration of your writing time. Constant interruptions disrupt your thinking and eat up your time for writing.

## Rule 3: Write first, edit later

During your designated writing time: Write! If you feel stuck, try writing whatever pops into your head for the first five minutes, or label your draft as a “cruddy first draft,” or write your thoughts as a letter to someone who you care about so you are not distracted by making it perfect. Just putting something down on paper will drive the creative process. Ignore your internal editor when it interferes—your task is to write without inhibition. The evaluative component, i.e., editing and polishing, should come later.

## Rule 4: Use triggers to develop a productive writing habit

Maintaining a concrete habit can shift your attitude towards writing and reduce anxiety about how to start, how to finish, and how to make your work flow. To ensure your habit develops, make use of triggers to kick off that automatic urge to write like taking a brisk walk before writing or making a pot of your favorite tea. Maybe you decide to write after your department’s weekly research seminar. Music can be an effective trigger too, as long as it doesn’t take too much of your attention. Spend some time determining a few effective triggers and strategically place these before your writing time to help you write routinely. Your goal should be to make writing a habit, like brushing your teeth, so even if you don’t feel like writing you still do it.

## Rule 5: Be accountable

Establishing any new habit is hard without accountability. How can you best ensure that you stick to it? You can work with rewards—allowing yourself to read a chapter in that new book you love, watch a short video, or have a second cup of coffee after completing your writing time. We have found it helpful to pair up with a writing buddy. For example, once you have a time established, you can text or email your colleague to say, “I’m starting,” and then contact them again when you are done. The simple act of communicating your habit can help you keep on course. You could also arrange to meet somewhere and write together, as long as you remain focused on the tasks at hand.

## Rule 6: Seek feedback and ask for what you want

It can be scary to share your drafts and elicit feedback, but writing effectively is not a solitary activity. Eventually someone is going to read what you wrote, and you can help make that experience a good one for your audience. You can and should elicit feedback from all sorts of people. Ask for feedback early and often from fellow graduate students or postdocs, other colleagues or collaborators, and research mentors, both within and outside of your discipline. Nonscientists can also provide valuable feedback about the clarity of your ideas. When you share a draft, give your reader some idea of where you are in the writing process and what kinds of things you would like them to focus on. For example, you might ask whether the first paragraph is clear, or what they think the major point is, or if the overall argument is persuasive. Many readers end up focusing on grammar, so let them know ahead of time if you want something more than copyediting. Ask your readers to prioritize their feedback, and give you their top three issues in detail. Good writing requires this iterative process, and each revision will refine your writing.

## Rule 7: Think about what you’re writing outside of your scheduled writing time

Sometimes the best thinking is done when you are otherwise occupied with a mundane task. Similarly, thinking about what you are working on outside your scheduled writing time can lead to more effective writing. Take time away from your writing to allow your mind to churn. Go to the animal facility to manage the murine colony, image cells on the confocal microscope, talk about your latest exciting result, etc. Use this time outside your scheduled writing time to mentally outline early drafts, deepen your argument, refine your hypothesis, etc. Most importantly, thinking about your writing can stimulate your desire to write.

## Rule 8: Practice, practice, practice

Just like any other skill, through practice you will become a better writer. Seek out opportunities to learn new approaches to be a more effective writer. For example, if you are currently working on a manuscript, learn more about that genre. As an example, see the Massive Open Online Course *Writing in the Sciences* [5] and these other sources for best practices for composing effective manuscripts [6–8]. Reading relentlessly will also help you to become a better writer, so reread those high-impact papers in your field and analyze the authors’ effectiveness at communicating the significance of their research. Do not limit yourself to scientific papers; read a variety of good writing. It is also critical to sharpen your writing skills through exploring writing style and elegance. We recommend reading a classic book on the topic, *Elements of Style* [9]. It can be helpful to practice engaging audiences by writing for diverse genres (i.e., manuscript, research plan for a proposal, lay abstract, commentary, blog, etc.), which will also keep your writing practice from becoming monotonous.

We also recommend that you surround yourself with a supportive community of other writers. Learn from these writers by offering to read and provide feedback on their writing. Asking for feedback and providing it to others will make you a more skilled editor, mentor, and writer.

## Rule 9: Manage your self-talk about writing

Be mindful of your self-talk about writing because negative thinking is detrimental to your writing practice and can squelch your writing. Make a conscious effort to silence your internal editor and redirect demoralizing messages (“There is too much to do. I don’t have enough time.”) to positive, hopeful thoughts (“I am going to make progress today by writing for one hour”). Remind yourself that even incremental progress will lead to something bigger with time.

## Rule 10: Reevaluate your writing practice often

Track your writing, perhaps by marking it off on a calendar, which can serve as a reminder when you have missed a few days. It is normal for your writing practice to have cyclic ups and downs. Don’t be discouraged; all writers must overcome writing resistance at some time or another. If your writing practice is not where you want it to be, think about what is holding you back (see Box 1), then develop strategies to overcome your resistance to writing (see Rules 1–10 and Box 2). Also, periodically accelerate your writing practice by joining a writing retreat or planning a weekend away to write. Truly, the only way to maintain your writing productivity is to keep writing.

**Box 2. Scientists can improve their written productivity using Rules 1–10 and advice from other writers**

Tips about writing from writers

“The scariest moment is always just before you start.”—Stephen King [10]

“Your desire to write grows with writing.”—Desiderius Erasmus [11]

“Write freely and as rapidly as possible and throw the whole thing on paper. Never correct or rewrite until the whole thing is done. Rewrite in process is usually found to be an excuse for not moving on. It also interferes with flow and rhythm which can only come from a kind of unconscious association with the material…”—John Steinbeck [12]

“Just write every day of your life. Read intensely. Then see what happens. Most of my friends who are put on that diet have very pleasant careers.”—Ray Bradbury [13]

“This is how you do it: You sit down at the keyboard and you put one word after another until it’s done. It’s that easy, and that hard.”—Neil Gaiman [14]

“Almost all good writing begins with terrible first efforts. You need to start somewhere.”—Anne Lamott [15]

“My ideas usually come not at my desk writing but in the midst of living.”—Anais Nin [16]

“Amateurs sit and wait for inspiration, the rest of us just get up and go to work.”—Stephen King [17]

“You can’t think yourself out of a writing block; you have to write yourself out of a thinking block.”—John Rogers [18]

“There is no failure unless one stops.”—Ray Bradbury [19]

## Conclusion

Writing is one of the most important activities that we as scientists engage in because it is critical to share our findings both within and beyond our research community. Use these 10 simple rules to write more and increase your impact as a scientist. But like the iterative process of writing, also evaluate your writing practice often (see Rule 10). Note that it may not be necessary to follow all the rules all the time; experiment and find what works for your writing practice. If you find your writing sessions are becoming less productive because you are facing new hurdles to writing (see Box 1), refine your writing practice using Rules 1–10.

## References

[1] Schimel Joshua. Writing Science: How to Write Papers that Get Cited and Proposals that Get Funded (Oxford University Press, USA, 2012), 4.

[2] Montgomery Scott L. The Chicago Guide to Communicating Science (University of Chicago Press, Chicago, IL, 2003), 41.

[3] The Eloquent Science Sites, Quotes from Experts on Effective Scientific Writing, 2009. Available from: http://eloquentscience.com/2009/08/quotes-from-experts-on-effective-scientific-writing/. [cited 2018 April 4].

[4] SMART Criteria. Wikipedia. Available from: https://en.wikipedia.org/wiki/SMART_criteria. [cited 2017 December 21].

[5] Sainani Kristin, Writing in the Sciences. Available from: https://lagunita.stanford.edu/courses/Medicine/SciWrite-SP/SelfPaced/about. [cited 2017 December 21].

[6] Zhang W. (2014) Ten Simple Rules for Writing Research Papers. PLoS Comput Biol 10(1): e1003453 10.1371/journal.pcbi.1003453 10.1371/journal.pcbi.1003453 24499936 PMC3907284

[7] Mensh B and K. Kording. (2017) Ten Simple Rules for Structuring Papers. PLoS Comput Biol 13(9): e1005619 10.1371/journal.pcbi.1005619 10.1371/journal.pcbi.1005619 28957311 PMC5619685

[8] Welch Gilbert H. (1999) Preparing Manuscripts for Submission to Medical Journals: The Paper Trail. Effective Clinical Practice. 2:131–137. 10538262

[9] Strunk, William Jr. The Elements of Style. Pearson Education Limited (England, 2014).

[10] King, Stephen. On Writing: A Memoir of the Craft. 10th Anniversary Edition (New York: Pocket Books, 2000), 274.

[11] Lilless M Shilling., and Fuller Linda K., editors. Dictionary of Quotations in Communications (Westport, Connecticut: Greenwood Press, 1997), 276.

[12] Quoted in George Plimpton, “John Steinbeck, The Art of Fiction No. 45 (Continued),” The Paris Review, 1975.

[13] Perrin, Timothy. (1986) “Ray Bradbury’s Nostalgia for the Future,” Writer’s Digest, February.

[14] The Neil Gaiman Journal Sites. Pens, Rules, Finishing Things and Why Stephin Merritt is Not Grouchy, 2004. Available from: journal.neilgaiman.com/2004/05/pens-rules-finishing-things-and-why.asp. [cited 2017 Sept. 9].

[15] Lamott Anne. Bird by Bird: Some Instruction on Writing and Life 1^st^ Edition (New York: Anchor Books, 1994), 25.

[16] Nin, Anais. The Diary of Anais Nin Volume 3 1939:1944. (San Diego, California: A Harvest/HBJ Book, 1969) Vol. 3, 29.

[17] King Stephen. On Writing: A Memoir of the Craft 10^th^ Anniversary Edition (New York: Pocket Books, 2000).

[18] The Kung Fu Monkey Sites. So Where the Hell Have you Been?, 2011. Available from: kfmonkey.blogspot.com/2011/06/so-where-hell-have-you-been.html. [cited 2017 September 9].

[19] Bradbury Ray. Zen of the Art of Writing: Releasing the Creative Genius Within You (New York: Bantam, 1992), 133.

